# The genome sequence of the cuckoo wrasse, *Labrus mixtus *Linnaeus 1758

**DOI:** 10.12688/wellcomeopenres.23063.1

**Published:** 2024-09-26

**Authors:** Belle Heaton, Rachel Brittain, Patrick Adkins, Kesella Scott-Somme, Joanna Harley, Marine Biological Association Genome Acquisition Lab, Darwin Tree of Life Barcoding collective, Wellcome Sanger Institute Tree of Life Management, Samples and Laboratory team, Wellcome Sanger Institute Scientific Operations: Sequencing Operations, Wellcome Sanger Institute Tree of Life Core Informatics team, Tree of Life Core Informatics collective, Darwin Tree of Life Consortium

**Affiliations:** 1 The Marine Biological Association, Plymouth, England, UK

**Keywords:** Labrus mixtus, cuckoo wrasse, genome sequence, chromosomal, Labriformes

## Abstract

We present a genome assembly from an individual *Labrus mixtus* (the cuckoo wrasse; Chordata; Actinopteri; Labriformes; Labridae). The genome sequence has a total length of 740.60 megabases. Most of the assembly is scaffolded into 24 chromosomal pseudomolecules. The mitochondrial genome has also been assembled and is 16.49 kilobases in length.

## Species taxonomy

Eukaryota; Opisthokonta; Metazoa; Eumetazoa; Bilateria; Deuterostomia; Chordata; Craniata; Vertebrata; Gnathostomata; Teleostomi; Euteleostomi; Actinopterygii; Actinopteri; Neopterygii; Teleostei; Osteoglossocephalai; Clupeocephala; Euteleosteomorpha; Neoteleostei; Eurypterygia; Ctenosquamata; Acanthomorphata; Euacanthomorphacea; Percomorphaceae; Eupercaria; Labriformes; Labridae; *Labrus*; *Labrus mixtus* Linnaeus 1758 (NCBI:txid508554).

## Background

*Labrus mixtus* is a colourful wrasse species, commonly found in inshore coastal waters of Northern Europe ( Quigley, 2009) to depths of up to 200 m ( Costello *et al.*, 1995; Gregory, 2003). They grow up to 35 cm in length and live to 13–17 years of age ( Darwall *et al.*, 1992; Matić-Skoko *et al.*, 2013). *Labrus mixtus* is distributed around the west and south coasts of the British Isles ( Heessen *et al.*, 2015; NBN Atlas Partnership, 2024). It occurs mainly in macroalga-dominated rocky reef habitats as well as seagrass beds ( Matić-Skoko *et al*., 2013). Wrasse species are opportunistic crusher feeders ( Gerking, 1994), breaking hard-shelled invertebrates with strong teeth in the jaws (for biting and rasping) and on the pharyngeal bones in the throat (for gripping and crushing) ( Liem & Sanderson, 1986). A study by Matić-Skoko *et al.* (2013) found that crustaceans and gastropods make up 68.7% of the diet for >70% of the fish.

*Labrus mixtus* belongs to the *Labridae* family and is a protogynous hermaphrodite. Females are sexually mature at 19 cm total length (TL), while all males mature at 29 cm TL, with sex changing from female to male at a length of 26 cm if no males are present in the area ( Matić-Skoko *et al*., 2013). More intensely-coloured males have more reproductive success ( Robertson, 1972), and this colour manifests as a dark blue head, bright orange body and fins, with bright blue markings overlaying the fins and body. Females and young (accessory) males have similar colouration, rose-pink to orange-red. However, females can be distinguished from males by the 2 to 3 dark spots interspersed with white on the rear dorsal and adjacent tail fin ( Gregory, 2003). Spawning season in the Atlantic is between May and July and occurs in pairs, unless accessory males are present ( Darwall *et al*., 1992). Sticky benthic eggs are deposited in nests (depressions in the sediment), which are both created and guarded by the males ( Riley *et al.*, 2017).

No significant fishery exists for this species, and it is only occasionally used as a commercial cleaner fish species ( Riley *et al.,* 2017). It is popular amongst recreational anglers, however, *L. mixtus* is currently listed as a species of least concern on the IUCN ( Pollard & Afonso, 2010). Here we present the first chromosomally complete genome sequence for *L. mixtus*, generated as part of the Darwin Tree of Life project.

## Genome sequence report

**Figure 1.**
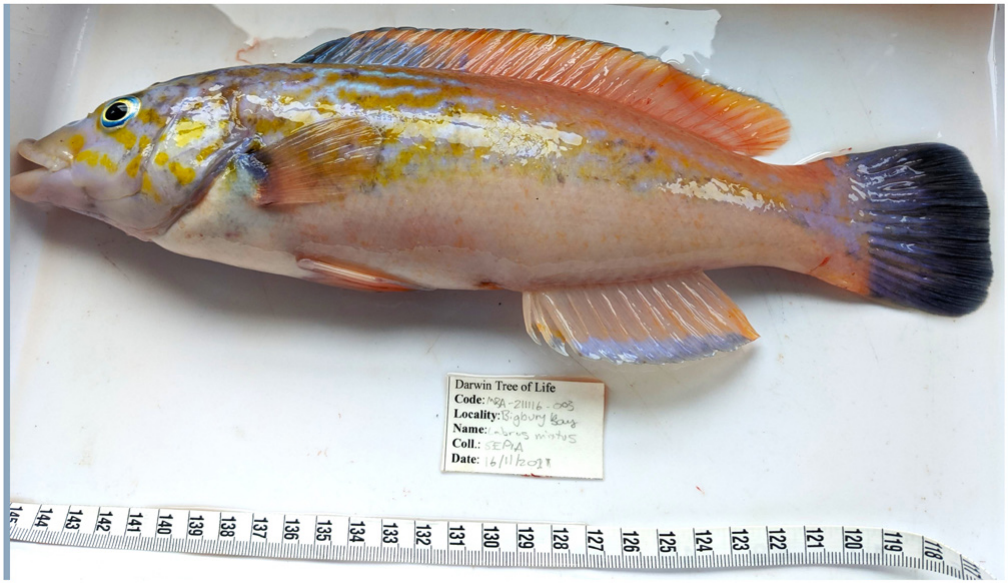
Photograph of the *Labrus mixtus* (fLabMix1) specimen used for genome sequencing.

The genome of an adult *Labrus mixtus* ( Figure 1) was sequenced using Pacific Biosciences single-molecule HiFi long reads, generating a total of 65.68 Gb (gigabases) from 7.06 million reads, providing approximately 35-fold coverage. Primary assembly contigs were scaffolded with chromosome conformation Hi-C data, which produced 94.77 Gb from 627.64 million reads, yielding an approximate coverage of 128-fold. Specimen and sequencing information is summarised in Table 1.

**Table 1.** Specimen and sequencing data for *Labrus mixtus*.

Project information			
**Study title**	Labrus mixtus (cuckoo wrasse)		
**Umbrella BioProject**	PRJEB63507		
**Species**	*Labrus mixtus*		
**BioSample**	SAMEA111562155		
**NCBI taxonomy ID**	508554		
Specimen information			
**Technology**	**ToLID**	**BioSample accession**	**Organism part**
**PacBio long read sequencing**	fLabMix1	SAMEA111562281	gill_animal
**Hi-C sequencing**	fLabMix1	SAMEA111562282	gill_animal
**RNA sequencing**	fLabMix1	SAMEA111562282	gill_animal
Sequencing information			
**Platform**	**Run accession**	**Read count**	**Base count (Gb)**
**Hi-C Illumina NovaSeq 6000**	ERR11606340	6.28e+08	94.77
**PacBio Sequel IIe**	ERR11641064	2.40e+06	23.96
**PacBio Revio**	ERR11809133	7.06e+06	65.68
**RNA Illumina NovaSeq 6000**	ERR12035199	5.79e+07	8.74

**Figure 2.**
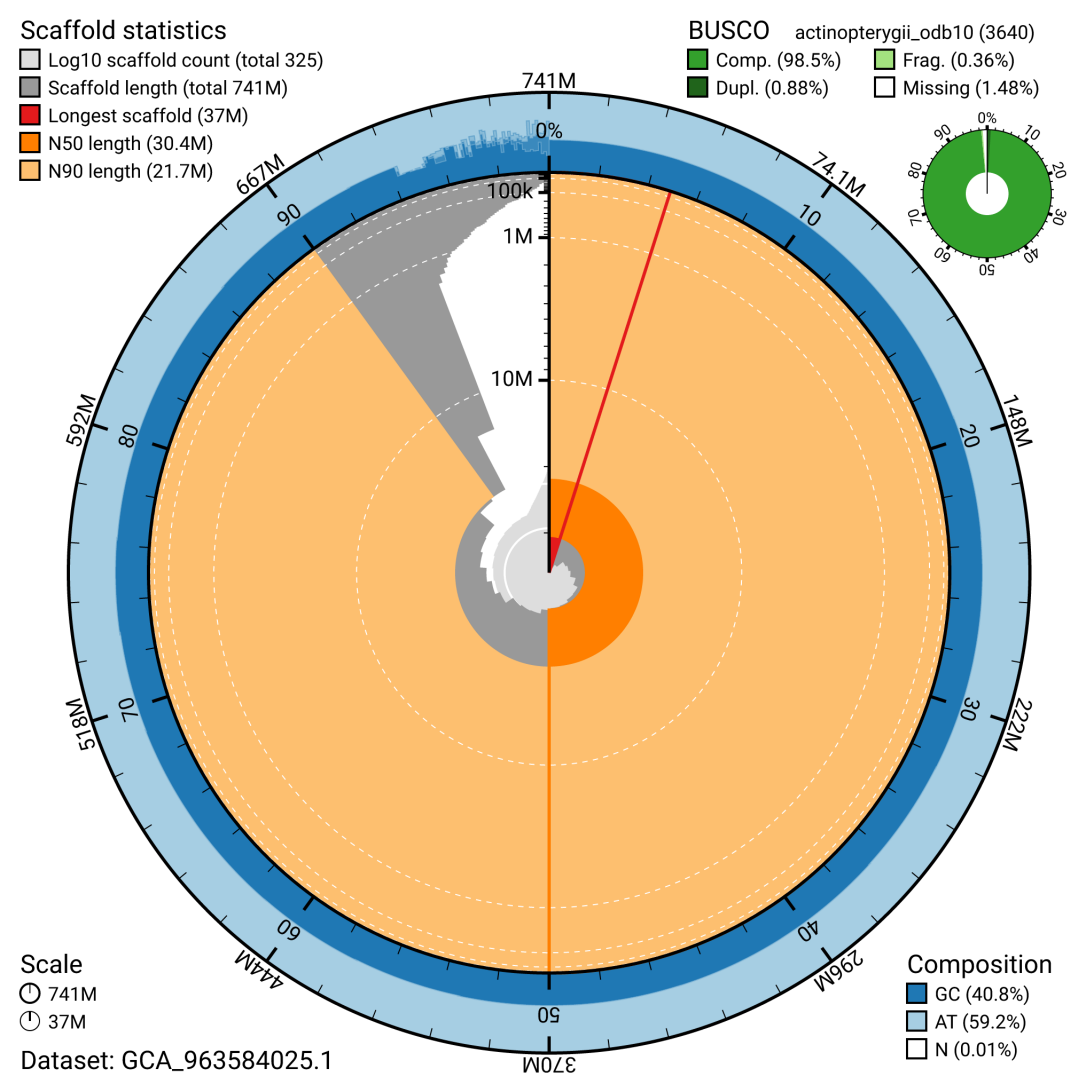
Genome assembly of *Labrus mixtus*, fLabMix1.1: metrics. The BlobToolKit snail plot shows N50 metrics and BUSCO gene completeness. The main plot is divided into 1,000 size-ordered bins around the circumference with each bin representing 0.1% of the 740,579,663 bp assembly. The distribution of scaffold lengths is shown in dark grey with the plot radius scaled to the longest scaffold present in the assembly (36,961,920 bp, shown in red). Orange and pale-orange arcs show the N50 and N90 scaffold lengths (30,403,003 and 21,671,947 bp), respectively. The pale grey spiral shows the cumulative scaffold count on a log scale with white scale lines showing successive orders of magnitude. The blue and pale-blue area around the outside of the plot shows the distribution of GC, AT and N percentages in the same bins as the inner plot. A summary of complete, fragmented, duplicated and missing BUSCO genes in the actinopterygii_odb10 set is shown in the top right. An interactive version of this figure is available at https://blobtoolkit.genomehubs.org/view/GCA_963584025.1/dataset/GCA_963584025.1/snail.

**Figure 3.**
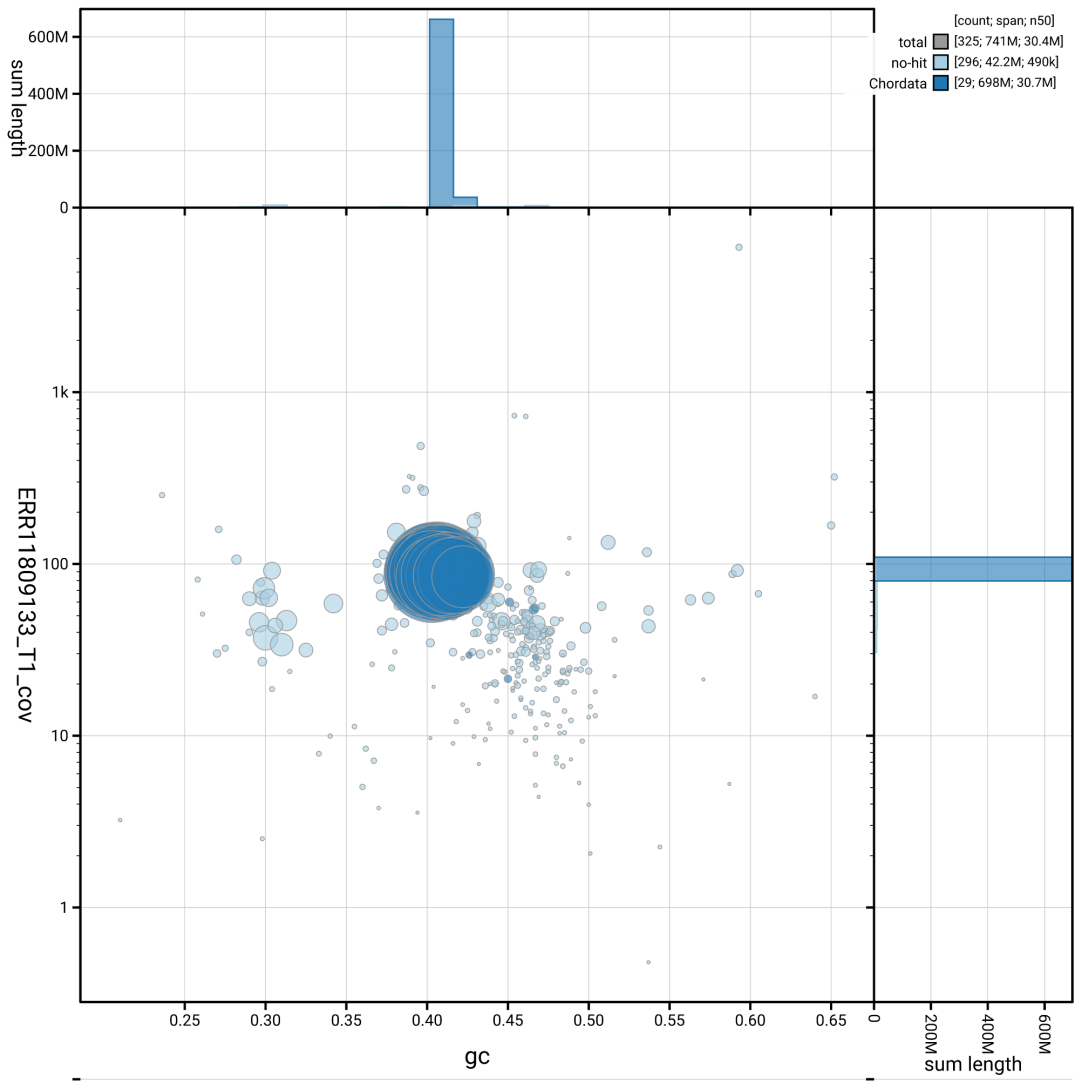
Genome assembly of *Labrus mixtus*, fLabMix1.1: Blob plot of base coverage against GC proportion for sequences in the assembly. Sequences are coloured by phylum. Circles are sized in proportion to sequence length. Histograms show the distribution of sequence length sum along each axis. An interactive version of this figure is available at https://blobtoolkit.genomehubs.org/view/GCA_963584025.1/dataset/GCA_963584025.1/blob.

**Figure 4.**
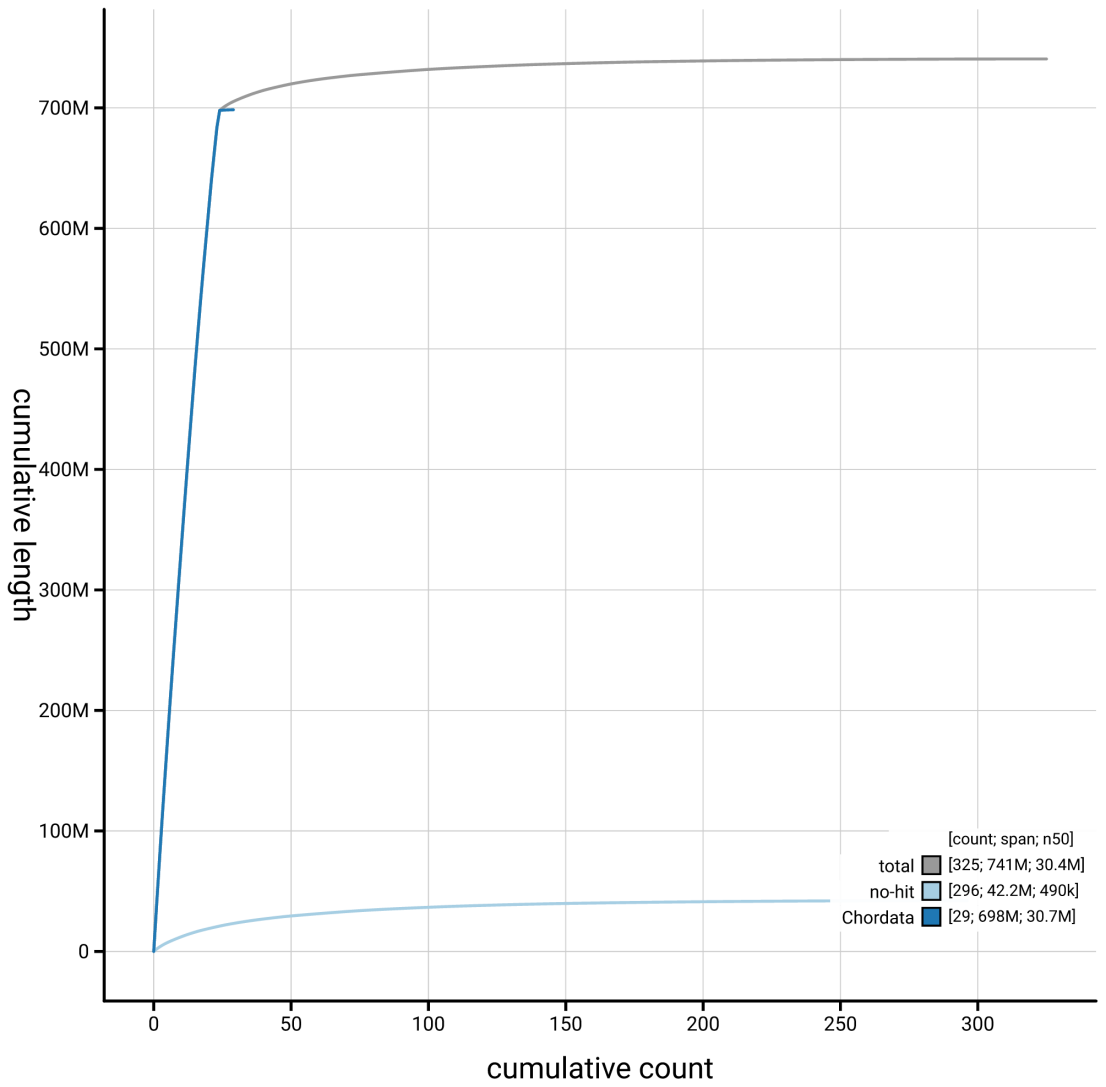
Genome assembly of *Labrus mixtus* fLabMix1.1: BlobToolKit cumulative sequence plot. The grey line shows cumulative length for all sequences. Coloured lines show cumulative lengths of sequences assigned to each phylum using the buscogenes taxrule. An interactive version of this figure is available at https://blobtoolkit.genomehubs.org/view/GCA_963584025.1/dataset/GCA_963584025.1/cumulative.

**Figure 5.**
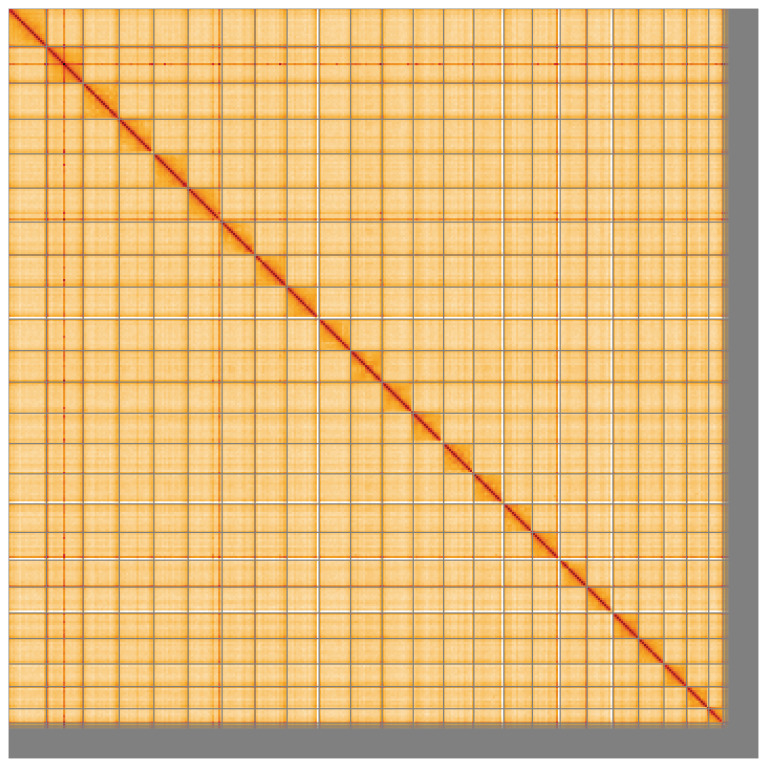
Genome assembly of *Labrus mixtus* fLabMix1.1: Hi-C contact map of the fLabMix1.1 assembly, visualised using HiGlass. Chromosomes are shown in order of size from left to right and top to bottom. An interactive version of this figure may be viewed at https://genome-note-higlass.tol.sanger.ac.uk/l/?d=Y4a1WR1GS7i8XWD2b1g7uQ.

Manual assembly curation corrected 18 missing joins or mis-joins, reducing the scaffold number by 1.52%, and increasing the scaffold N50 by 0.95%. The final assembly has a total length of 740.60 Mb in 324 sequence scaffolds, with 570 gaps, and a scaffold N50 of 30.4 Mb ( Table 2). The snail plot in Figure 2 provides a summary of the assembly statistics, while the distribution of assembly scaffolds on GC proportion and coverage is shown in Figure 3. The cumulative assembly plot in Figure 4 shows curves for subsets of scaffolds assigned to different phyla. Most (94.23%) of the assembly sequence was assigned to 24 chromosomal-level scaffolds. Chromosome-scale scaffolds confirmed by the Hi-C data are named in order of size ( Figure 5; Table 3). While not fully phased, the assembly deposited is of one haplotype. Contigs corresponding to the second haplotype have also been deposited. The mitochondrial genome was also assembled and can be found as a contig within the multifasta file of the genome submission.

**Table 2.** Genome assembly data for *Labrus mixtus*, fLabMix1.1.

Genome assembly		
Assembly name	fLabMix1.1	
Assembly accession	GCA_963584025.1	
*Accession of alternate haplotype*	*GCA_963583885.1*	
Span (Mb)	740.60	
Number of contigs	895	
Contig N50 length (Mb)	2.7	
Number of scaffolds	324	
Scaffold N50 length (Mb)	30.4	
Longest scaffold (Mb)	36.96	
Assembly metrics *		*Benchmark*
Consensus quality (QV)	59.5	*≥ 50*
*k*-mer completeness	100.0%	*≥ 95%*
BUSCO **	C:98.5%[S:97.6%,D:0.9%], F:0.4%,M:1.1%,n:3,640	*C ≥ 95%*
Percentage of assembly mapped to chromosomes	94.23%	*≥ 95%*
Sex chromosomes	Not identified	*localised homologous pairs*
Organelles	Mitochondrial genome: 16.49 kb	*complete single alleles*

* Assembly metric benchmarks are adapted from column VGP-2020 of “Table 1: Proposed standards and metrics for defining genome assembly quality” from Rhie *et al.* (2021). ** BUSCO scores based on the actinopterygii_odb10 BUSCO set using version 5.4.3. C = complete [S = single copy, D = duplicated], F = fragmented, M = missing, n = number of orthologues in comparison. A full set of BUSCO scores is available at https://blobtoolkit.genomehubs.org/view/Labrus_mixtus/dataset/GCA_963584025.1/busco.

**Table 3.** Chromosomal pseudomolecules in the genome assembly of *Labrus mixtus*, fLabMix1.

INSDC accession	Name	Length (Mb)	GC%
OY757284.1	1	36.96	40.5
OY757285.1	2	35.93	40.5
OY757286.1	3	35.2	40.5
OY757287.1	4	33.82	40.5
OY757288.1	5	33.48	41.0
OY757289.1	6	33.13	40.5
OY757290.1	7	32.15	41.0
OY757291.1	8	31.33	40.5
OY757292.1	9	31.24	41.0
OY757293.1	10	30.83	41.0
OY757294.1	11	30.72	41.0
OY757295.1	12	30.4	40.5
OY757296.1	13	29.66	40.0
OY757297.1	14	29.55	40.5
OY757298.1	15	29.32	41.0
OY757299.1	16	27.88	41.0
OY757300.1	17	27.05	40.5
OY757301.1	18	26.31	41.5
OY757302.1	19	25.3	40.5
OY757303.1	20	24.95	41.0
OY757304.1	21	24.81	41.0
OY757305.1	22	22.08	42.0
OY757306.1	23	21.67	41.5
OY757307.1	24	14.07	42.0
OY757308.1	MT	0.02	46.5

The estimated Quality Value (QV) of the final assembly is 59.5 with *k*-mer completeness of 100.0%, and the assembly has a BUSCO v5.4.3 completeness of 98.5% (single = 97.6%, duplicated = 0.9%), using the actinopterygii_odb10 reference set ( *n* = 3,640).

Metadata for specimens, BOLD barcode results, spectra estimates, sequencing runs, contaminants and pre-curation assembly statistics are given at https://links.tol.sanger.ac.uk/species/508554.

## Methods

### Sample acquisition

A *Labrus mixtus* specimen (specimen ID MBA-211116-003A, ToLID fLabMix1) was collected from Bigbury Bay, English Channel, UK (latitude 50.27, longitude –3.97) on 2021-11-16. The specimen was taken from its habitat of sand and broken shell using an otter trawl deployed from RV Sepia. The specimen was identified by Rachel Brittain (Marine Biological Association), based on gross morphology. The fish was first anesthetised and then overdosed using Aquased (2-phenoxyethanol). Destruction of the brain was used as a secondary method to ensure the animal was deceased before tissue sampling took place as in accordance with Schedule 1 methodology under the home office licence. Samples taken from the animal were preserved on dry ice.

The initial identification was verified by an additional DNA barcoding process according to the framework developed by Twyford *et al*. (2024). A small sample was dissected from the specimens and stored in ethanol, while the remaining parts of the specimen were shipped on dry ice to the Wellcome Sanger Institute (WSI). The tissue was lysed, the COI marker region was amplified by PCR, and amplicons were sequenced and compared to the BOLD database, confirming the species identification ( Crowley *et al.*, 2023). Following whole genome sequence generation, the relevant DNA barcode region was also used alongside the initial barcoding data for sample tracking at the WSI ( Twyford *et al.*, 2024). The standard operating procedures for Darwin Tree of Life barcoding have been deposited on protocols.io ( Beasley *et al.*, 2023).

### Nucleic acid extraction

The workflow for high molecular weight (HMW) DNA extraction at the WSI Tree of Life Core Laboratory includes a sequence of core procedures: sample preparation and homogenisation, DNA extraction, fragmentation and purification. Detailed protocols are available on protocols.io ( Denton *et al.*, 2023b). The fLabMix1 sample was prepared for DNA extraction by weighing and dissecting it on dry ice ( Jay *et al.*, 2023). Tissue from the gill was homogenised using a PowerMasher II tissue disruptor ( Denton *et al.*, 2023a).

HMW DNA was extracted in the WSI Scientific Operations core using the Automated MagAttract v2 protocol ( Oatley *et al.*, 2023). The DNA was sheared into an average fragment size of 12–20 kb in a Megaruptor 3 system ( Bates *et al.*, 2023). Sheared DNA was purified by solid-phase reversible immobilisation, using AMPure PB beads to eliminate shorter fragments and concentrate the DNA ( Strickland *et al.*, 2023). The concentration of the sheared and purified DNA was assessed using a Nanodrop spectrophotometer and Qubit Fluorometer using the Qubit dsDNA High Sensitivity Assay kit. Fragment size distribution was evaluated by running the sample on the FemtoPulse system.

RNA was extracted from gill tissue of fLabMix1 in the Tree of Life Laboratory at the WSI using the RNA Extraction: Automated MagMax™ *mir*Vana protocol ( do Amaral *et al.*, 2023). The RNA concentration was assessed using a Nanodrop spectrophotometer and a Qubit Fluorometer using the Qubit RNA Broad-Range Assay kit. Analysis of the integrity of the RNA was done using the Agilent RNA 6000 Pico Kit and Eukaryotic Total RNA assay.

### Library preparation and sequencing

Pacific Biosciences HiFi circular consensus DNA sequencing libraries were constructed according to the manufacturers’ instructions. Poly(A) RNA-Seq libraries were constructed using the NEB Ultra II RNA Library Prep kit. DNA and RNA sequencing was performed by the Scientific Operations core at the WSI on Pacific Biosciences Revio (HiFi) and Illumina NovaSeq 6000 (RNA-Seq) instruments.

Hi-C data were generated from gill tissue of fLabMix1, using the Arima-HiC v2 kit. In brief, frozen tissue (–80 °C) was fixed, and the DNA crosslinked using a TC buffer containing formaldehyde. The crosslinked DNA was then digested using a restriction enzyme master mix. The 5’-overhangs were then filled in and labelled with a biotinylated nucleotide and proximally ligated. The biotinylated DNA construct was fragmented to a fragment size of 400 to 600 bp using a Covaris E220 sonicator. The DNA was then enriched, barcoded, and amplified using the NEBNext Ultra II DNA Library Prep Kit, following manufacturers’ instructions. The Hi-C sequencing was performed using paired-end sequencing with a read length of 150 bp on an Illumina NovaSeq 6000 instrument.

### Genome assembly, curation and evaluation

***Assembly***

The HiFi reads were first assembled using Hifiasm ( Cheng *et al.*, 2021) with the --primary option. Haplotypic duplications were identified and removed using purge_dups ( Guan *et al.*, 2020). The Hi-C reads were mapped to the primary contigs using bwa-mem2 ( Vasimuddin *et al.*, 2019). The contigs were further scaffolded using the provided Hi-C data ( Rao *et al.*, 2014) in YaHS ( Zhou *et al.*, 2023) using the --break option. The scaffolded assemblies were evaluated using Gfastats ( Formenti *et al.*, 2022), BUSCO ( Manni *et al.*, 2021) and MERQURY.FK ( Rhie *et al.*, 2020).

The mitochondrial genome was assembled using MitoHiFi ( Uliano-Silva *et al.*, 2023), which runs MitoFinder ( Allio *et al.*, 2020) and uses these annotations to select the final mitochondrial contig and to ensure the general quality of the sequence.

***Assembly curation***

The assembly was decontaminated using the Assembly Screen for Cobionts and Contaminants (ASCC) pipeline (article in preparation). Flat files and maps used in curation were generated in TreeVal ( Pointon *et al.*, 2023). Manual curation was primarily conducted using PretextView ( Harry, 2022), with additional insights provided by JBrowse2 ( Diesh *et al.*, 2023) and HiGlass ( Kerpedjiev *et al.*, 2018). Scaffolds were visually inspected and corrected as described by Howe *et al*. (2021). Any identified contamination, missed joins, and mis-joins were corrected, and duplicate sequences were tagged and removed. The curation process is documented at https://gitlab.com/wtsi-grit/rapid-curation (article in preparation).

***Evaluation of the final assembly***

The final assembly was post-processed and evaluated using the three Nextflow ( Di Tommaso *et al.*, 2017) DSL2 pipelines: sanger-tol/readmapping ( Surana *et al.*, 2023a), sanger-tol/genomenote ( Surana *et al.*, 2023b), and sanger-tol/blobtoolkit ( Muffato *et al.*, 2024). The readmapping pipeline aligns the Hi-C reads using bwa-mem2 ( Vasimuddin *et al.*, 2019) and combines the alignment files with SAMtools ( Danecek *et al.*, 2021). The genomenote pipeline converts the Hi-C alignments into a contact map using BEDTools ( Quinlan & Hall, 2010) and the Cooler tool suite ( Abdennur & Mirny, 2020). The contact map is visualised in HiGlass ( Kerpedjiev *et al.*, 2018). This pipeline also generates assembly statistics using the NCBI datasets report ( Sayers *et al.*, 2024), computes *k*-mer completeness and QV consensus quality values with FastK and MERQURY.FK, and runs BUSCO ( Manni *et al.*, 2021) to assess completeness.

The blobtoolkit pipeline is a Nextflow port of the previous Snakemake Blobtoolkit pipeline ( Challis *et al.*, 2020). It aligns the PacBio reads in SAMtools and minimap2 ( Li, 2018) and generates coverage tracks for regions of fixed size. In parallel, it queries the GoaT database ( Challis *et al.*, 2023) to identify all matching BUSCO lineages to run BUSCO ( Manni *et al.*, 2021). For the three domain-level BUSCO lineages, the pipeline aligns the BUSCO genes to the UniProt Reference Proteomes database ( Bateman *et al.*, 2023) with DIAMOND ( Buchfink *et al.*, 2021) blastp. The genome is also split into chunks according to the density of the BUSCO genes from the closest taxonomic lineage, and each chunk is aligned to the UniProt Reference Proteomes database with DIAMOND blastx. Genome sequences without a hit are chunked with seqtk and aligned to the NT database with blastn ( Altschul *et al.*, 1990). The blobtools suite combines all these outputs into a blobdir for visualisation.

The genome assembly and evaluation pipelines were developed using nf-core tooling ( Ewels *et al.*, 2020) and MultiQC ( Ewels *et al.*, 2016), relying on the Conda package manager, the Bioconda initiative ( Grüning *et al.*, 2018), the Biocontainers infrastructure ( da Veiga Leprevost *et al.*, 2017), as well as the Docker ( Merkel, 2014) and Singularity ( Kurtzer *et al.*, 2017) containerisation solutions.

Table 4 contains a list of relevant software tool versions and sources.

**Table 4.** Software tools: versions and sources.

Software tool	Version	Source
BEDTools	2.30.0	https://github.com/arq5x/bedtools2
BLAST	2.14.0	ftp://ftp.ncbi.nlm.nih.gov/blast/executables/blast+/
BlobToolKit	4.3.7	https://github.com/blobtoolkit/blobtoolkit
BUSCO	5.4.3 and 5.5.0	https://gitlab.com/ezlab/busco
bwa-mem2	2.2.1	https://github.com/bwa-mem2/bwa-mem2
Cooler	0.8.11	https://github.com/open2c/cooler
DIAMOND	2.1.8	https://github.com/bbuchfink/diamond
fasta_windows	0.2.4	https://github.com/tolkit/fasta_windows
FastK	427104ea91c78c3b8b8b49f1a7d6bbeaa869ba1c	https://github.com/thegenemyers/FASTK
Gfastats	1.3.6	https://github.com/vgl-hub/gfastats
GoaT CLI	0.2.5	https://github.com/genomehubs/goat-cli
Hifiasm	0.19.8-r603	https://github.com/chhylp123/hifiasm
HiGlass	44086069ee7d4d3f6f3f0012569789ec138f42b84aa44357826c0b6753eb28de	https://github.com/higlass/higlass
Merqury.FK	d00d98157618f4e8d1a9190026b19b471055b22e	https://github.com/thegenemyers/MERQURY.FK
MitoHiFi	3	https://github.com/marcelauliano/MitoHiFi
MultiQC	1.14, 1.17, and 1.18	https://github.com/MultiQC/MultiQC
NCBI Datasets	15.12.0	https://github.com/ncbi/datasets
Nextflow	23.04.0-5857	https://github.com/nextflow-io/nextflow
PretextView	0.2	https://github.com/sanger-tol/PretextView
purge_dups	1.2.5	https://github.com/dfguan/purge_dups
samtools	1.16.1, 1.17, and 1.18	https://github.com/samtools/samtools
sanger-tol/ascc	-	https://github.com/sanger-tol/ascc
sanger-tol/genomenote	1.1.1	https://github.com/sanger-tol/genomenote
sanger-tol/readmapping	1.2.1	https://github.com/sanger-tol/readmapping
Seqtk	1.3	https://github.com/lh3/seqtk
Singularity	3.9.0	https://github.com/sylabs/singularity
TreeVal	1.0.0	https://github.com/sanger-tol/treeval
YaHS	1.2a.2	https://github.com/c-zhou/yahs

### Wellcome Sanger Institute – Legal and Governance

The materials that have contributed to this genome note have been supplied by a Darwin Tree of Life Partner. The submission of materials by a Darwin Tree of Life Partner is subject to the **‘Darwin Tree of Life Project Sampling Code of Practice’**, which can be found in full on the Darwin Tree of Life website here. By agreeing with and signing up to the Sampling Code of Practice, the Darwin Tree of Life Partner agrees they will meet the legal and ethical requirements and standards set out within this document in respect of all samples acquired for, and supplied to, the Darwin Tree of Life Project. 

Further, the Wellcome Sanger Institute employs a process whereby due diligence is carried out proportionate to the nature of the materials themselves, and the circumstances under which they have been/are to be collected and provided for use. The purpose of this is to address and mitigate any potential legal and/or ethical implications of receipt and use of the materials as part of the research project, and to ensure that in doing so we align with best practice wherever possible. The overarching areas of consideration are:

• Ethical review of provenance and sourcing of the material

• Legality of collection, transfer and use (national and international) 

Each transfer of samples is further undertaken according to a Research Collaboration Agreement or Material Transfer Agreement entered into by the Darwin Tree of Life Partner, Genome Research Limited (operating as the Wellcome Sanger Institute), and in some circumstances other Darwin Tree of Life collaborators.

## Data Availability

European Nucleotide Archive: *Labrus mixtus* (cuckoo wrasse). Accession number PRJEB63507; https://identifiers.org/ena.embl/PRJEB63507 ( Wellcome Sanger Institute, 2023). The genome sequence is released openly for reuse. The *Labrus mixtus* genome sequencing initiative is part of the Darwin Tree of Life (DToL) project. All raw sequence data and the assembly have been deposited in INSDC databases. The genome will be annotated using available RNA-Seq data and presented through the Ensembl pipeline at the European Bioinformatics Institute. Raw data and assembly accession identifiers are reported in Table 1 and Table 2.

## Author information

Members of the Marine Biological Association Genome Acquisition Lab are listed here: https://doi.org/10.5281/zenodo.8382513.

Members of the Darwin Tree of Life Barcoding collective are listed here: https://doi.org/10.5281/zenodo.12158331

Members of the Wellcome Sanger Institute Tree of Life Management, Samples and Laboratory team are listed here: https://doi.org/10.5281/zenodo.12162482.

Members of Wellcome Sanger Institute Scientific Operations: Sequencing Operations are listed here: https://doi.org/10.5281/zenodo.12165051.

Members of the Wellcome Sanger Institute Tree of Life Core Informatics team are listed here: https://doi.org/10.5281/zenodo.12160324.

Members of the Tree of Life Core Informatics collective are listed here: https://doi.org/10.5281/zenodo.12205391.

Members of the Darwin Tree of Life Consortium are listed here: https://doi.org/10.5281/zenodo.4783558.
