# Supplementary material for: The genome sequence of the cuckoo wrasse, Labrus mixtus Linnaeus 1758
Source: Wellcome Open Res. 2024 Sep 26;9:549. [Version 1] doi: 10.12688/wellcomeopenres.23063.1 (PMC11561382; doi:10.12688/wellcomeopenres.23063.1)
Supplement: Peer review history

# Reviewer report: Reviewer response for version 1

**DOI:** 10.21956/wellcomeopenres.25399.r102857

**Published:** 2024-11-13

**Authors:** Richard Coleman

**Affiliations:** 1 University of Miami, Coral Gables, Florida, USA

This aims to describe the process for generating the genome sequence for the cuckoo wrasse, *Laburs mixtus*. The authors have provide a detailed job of describing the methods to generate the genome and evaluate the final assembly. A few minor notes:

 Figure 3 - it is unclear what the y-axis represents

 Figure 5 - there are no axes labels so difficult to interpret. 

 Outside of those corrects I encourage approval of this manuscript.

Are sufficient details of methods and materials provided to allow replication by others?

Yes

Is the rationale for creating the dataset(s) clearly described?

Yes

Are the datasets clearly presented in a useable and accessible format?

Partly

Are the protocols appropriate and is the work technically sound?

Yes

Reviewer Expertise:

Fish ecology and evolution

I confirm that I have read this submission and believe that I have an appropriate level of expertise to confirm that it is of an acceptable scientific standard.

# Reviewer report: Reviewer response for version 1

**DOI:** 10.21956/wellcomeopenres.25399.r102859

**Published:** 2024-10-23

**Authors:** Rohit Kolora

**Affiliations:** 1 Universitat Leipzig (Ringgold ID: 9180), Leipzig, Saxony, Germany

In the manuscript titled "The genome sequence of the cuckoo wrasse, *Labrus mixtus *Linnaeus 1758", Heaton et al. present the first chromosome level assembly of *L. mixtus. *They report the methods used to generate the assembly and its metrics. The manuscript is concise and to the point. However, it would be good to provide the rationale for generating the genome - it neither is in the IUCN list nor a model of interest to a particular phenotype, is it a good representative of its ecosystem due to its higher prevalence, is there any data of it being affected due to human intervention.

 For reproducing the study, the parameters used to generate the assembly have not been provided. If default parameters were used, please state it.

Are sufficient details of methods and materials provided to allow replication by others?

Partly

Is the rationale for creating the dataset(s) clearly described?

Partly

Are the datasets clearly presented in a useable and accessible format?

Yes

Are the protocols appropriate and is the work technically sound?

Yes

Reviewer Expertise:

Genomics, Genetics, Comparative biology, Bioinformatics

I confirm that I have read this submission and believe that I have an appropriate level of expertise to confirm that it is of an acceptable scientific standard.
